# Sirtinol Treatment Reduces Inflammation in Human Dermal Microvascular Endothelial Cells

**DOI:** 10.1371/journal.pone.0024307

**Published:** 2011-09-12

**Authors:** Angela Orecchia, Claudia Scarponi, Francesca Di Felice, Elisa Cesarini, Simona Avitabile, Antonello Mai, Maria Luisa Mauro, Valentina Sirri, Giovanna Zambruno, Cristina Albanesi, Giorgio Camilloni, Cristina M. Failla

**Affiliations:** 1 Molecular and Cell Biology Laboratory, IDI-IRCCS, Rome, Italy; 2 Experimental Immunology Laboratory, IDI-IRCCS, Rome, Italy; 3 Department of Biology and Biotechnology ‘C. Darwin’, University di Roma La Sapienza, Rome, Italy; 4 Department of Drug Chemistry and Technologies, Pasteur Institute, Cenci Bolognetti Foundation, University di Roma La Sapienza, Rome, Italy; 5 RNA Biology, FRE3402 CNRS, Université Pierre et Marie Curie, Paris, France; 6 Istituto di Biologia e Patologia Molecolari, CNR, Rome, Italy; Johns Hopkins School of Medicine, United States of America

## Abstract

Histone deacetylases (HDAC) are key enzymes in the epigenetic control of gene expression. Recently, inhibitors of class I and class II HDAC have been successfully employed for the treatment of different inflammatory diseases such as rheumatoid arthritis, colitis, airway inflammation and asthma. So far, little is known so far about a similar therapeutic effect of inhibitors specifically directed against sirtuins, the class III HDAC. In this study, we investigated the expression and localization of endogenous sirtuins in primary human dermal microvascular endothelial cells (HDMEC), a cell type playing a key role in the development and maintenance of skin inflammation. We then examined the biological activity of sirtinol, a specific sirtuin inhibitor, in HDMEC response to pro-inflammatory cytokines. We found that, even though sirtinol treatment alone affected only long-term cell proliferation, it diminishes HDMEC inflammatory responses to tumor necrosis factor (TNF)α and interleukin (IL)-1β. In fact, sirtinol significantly reduced membrane expression of adhesion molecules in TNFã- or IL-1β-stimulated cells, as well as the amount of CXCL10 and CCL2 released by HDMEC following TNFα treatment. Notably, sirtinol drastically decreased monocyte adhesion on activated HDMEC. Using selective inhibitors for Sirt1 and Sirt2, we showed a predominant involvement of Sirt1 inhibition in the modulation of adhesion molecule expression and monocyte adhesion on activated HDMEC. Finally, we demonstrated the *in vivo* expression of Sirt1 in the dermal vessels of normal and psoriatic skin. Altogether, these findings indicated that sirtuins may represent a promising therapeutic target for the treatment of inflammatory skin diseases characterized by a prominent microvessel involvement.

## Introduction

Sirtuins, the class III NAD^+^-dependent deacetylases, are emerging regulators of several biological processes [1]. Their initially-investigated activity was as mediators of the increased life-span that follows calorie restriction in yeast [2], but recent data indicate their involvement in aging, genomic stability, tumorigenesis, and metabolic diseases [3]. In mammals, seven sirtuins have been described, with Sirt1 being the closest homologue to the yeast Sir2 [4]. Therefore, most studies have been focused on Sirt1 whereas the other six sirtuins have been less investigated. Sirt1 was found to inhibit cellular senescence induced by DNA damage and oxidative stress [5], and to increase upon caloric restriction or nutrient starvation [6]. At the molecular level, Sirt1 plays a role in transcriptional and post-transcriptional regulation of gene expression through deacetylation of histones and non-histone proteins, and novel targets of Sirt1 are continuously discovered [1]. Regarding vascular biology, Sirt1 is a key mediator of angiogenic signaling in postnatal neovascularization both *in vitro* and in models of zebrafish and mouse [7]. Sirt1 inhibition induces a premature senescence-like phenotype in cultured umbilical vein endothelial cells (HUVEC), in parallel with increased p53 acetylation [8], and in porcine aortic endothelial cells, as a consequence of the deregulation of the LKB1-AMPK pathway [9]. Moreover, Sirt1 plays a role in regulating endothelial nitric oxide and endothelium-dependent vascular tone by deacetylating nitric oxide synthase [10].

Even though initially developed as anti-cancer agents, class I and II HDAC inhibitors have shown therapeutic utility in the treatment of chronic immune and inflammatory disorders such as rheumatoid arthritis [11], colitis [12], airway inflammation and asthma [13]. So far, little is known so far about a possible therapeutic effect of inhibitors specifically directed against sirtuins, but recent findings indicate that inhibition of Sirt1 attenuates antigen-induced airway inflammation and hyperresponsiveness [14].

Morphological and functional alterations of microvessels are hallmark features of inflammatory disorders. In fact, microvascular endothelial cells are highly-specialized regulators of both tissue homeostasis and pathological process of inflammation [15], [16]. In response to inflammatory cytokines, microvascular endothelial cells become activated, display increased leakiness, secrete additional inflammatory chemokines, enhance leukocyte chemotaxis and adhesiveness to vessel wall, thus favoring leukocyte extravasation, distribution, and homing to the specific tissue. Specific differences exist between endothelial cells of the microvasculature and cells lining large blood vessels, and between microvascular endothelial cells from different tissues, including differences in secreted products, in cell adhesion molecule expression, in the molecular response to cytokines [17], [18], [19], [20]. Thus, evaluation of inflammation mechanisms in selected tissues should be performed directly on tissue-derived microvascular endothelial cells rather than on large vessel-derived endothelium. In this study, we analyzed the expression and localization of sirtuins in primary human dermal microvascular endothelial cells (HDMEC), and underlined the effectiveness of sirtuin inhibition in the modulation of flogistic responses of HDMEC activated with pro-inflammatory cytokines.

## Results

### Sirtuin expression pattern

**Figure 1:**
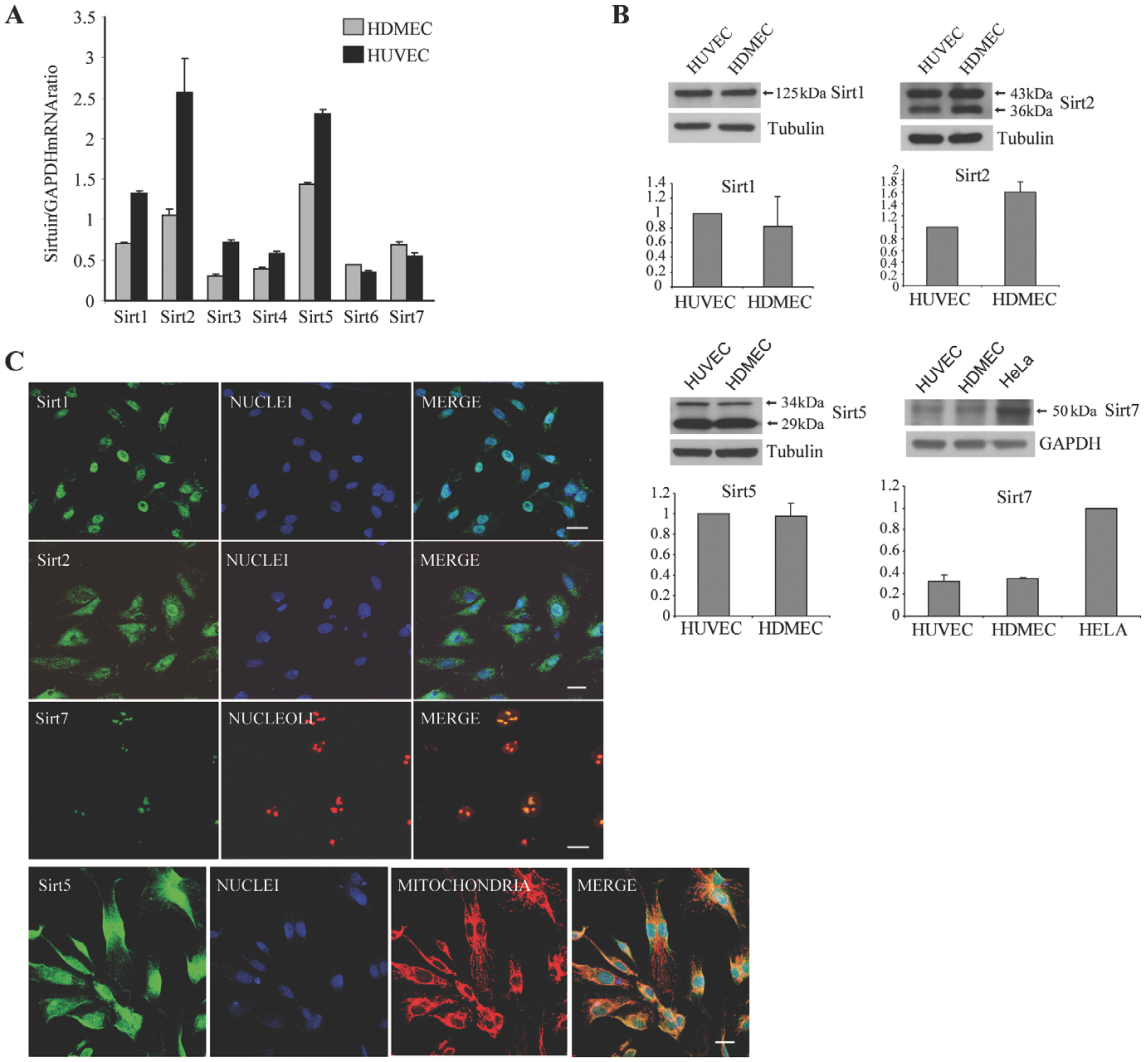
Sirtuin expression in endothelial cells. (A) Semi-quantitative RT-PCR of sirtuin expression. Bars refer to mRNA amounts relative to GAPDH mRNA. Results are shown as the mean ± s.d. of at least three experiments. (B) Western blotting of four sirtuins, indicated by arrows, the molecular weights of which are given in kDa. A representative experiment is reported. Tubulin and GAPDH were used as loading controls. The densitometric analysis of three different experiments is shown as the mean ± s.d. HUVEC values for the first three sirtuins and HELA value for Sirt7 were used as reference and reported as 1 (C) Sirtuins were stained with the corresponding primary antibody (left panel). HDMEC were also immunostained with a nuclear or a nucleolus marker (middle panels), or with a mitochondria marker (Sirt5, third panel from the left). Merged pictures in the right panels. Each panel is a total projection of a confocal stack of images; bars  =  20 µm.

To analyze the mRNA expression pattern of the seven mammalian sirtuins in HDMEC, semi-quantitative RT-PCR analyses was performed and showed that Sirt5, Sirt2, and Sirt1 were highly expressed, followed by Sirt7 (Fig. 1A). A similar pattern of sirtuin expression was present in HUVEC, even though a higher amount of sirtuin-encoding mRNA was generally detected, and two mitochondrial sirtuins [21], Sirt3 and Sirt4, were slightly more expressed than Sirt7 (Fig. 1A). HDMEC sirtuin expression pattern was cell-type specific, as it differed from those of other skin cells (Data S1), such as keratinocytes, fibroblasts, and melanocytes (Fig. S1A).

Protein expression of the four sirtuins more represented in HDMEC at the RNA level was also analyzed (Fig. 1B). Protein amount of the four sirtuins was similar between HDMEC and HUVEC. Moreover, differently from mRNA, Sirt2 protein amount was slightly higher in HDMEC compared to HUVEC, probably reflecting a different posttranscriptional regulation. The two known isoforms of Sirt2 and Sirt5 were distinguished, even if their relative amount varied among cell extract preparations (see Fig. 1B and Fig. S1B). The 50 kDa Sirt7 protein was also present, barely detectable in endothelial cells compared to HeLa cells, used as a positive control [22].

To investigate cellular localization of these four sirtuins, confocal microscopy was performed on HDMEC. As expected, Sirt1 was detected in the nucleus, whereas Sirt2 was equally present in the cytoplasm and nucleus. Sirt5 was detected both in mitochondria and nuclei, and Sirt7 co-localized in the nucleoli [22], [23] with the upstream binding transcription factor (UBF) (Fig. 1C). We evaluated Sirt2 and Sirt5 localization also on extracts prepared through differential cell lysis (Data S1) and confirmed that these sirtuins were present in both the cytoplasmic and nuclear compartments (Fig. S1B).

### Effect of sirtinol on cell growth and histone acetylation in HDMEC

Sirtinol, 2-[(2-Hydroxynaphthalen-1-ylmethylene)amino]-N-(1-phenethyl)benzamide, is a selective inhibitor of sirtuin enzymatic activity that does not affect class I or class II HDACs [24], [25]. As it was previously reported that sirtinol interferes with HUVEC growth [7], we verified sirtinol effectiveness in our cell type. HDMEC were treated for 18 hours with different concentrations of the inhibitor. Fetal calf serum, as a proliferation stimulus, was then added and cells were cultured for 9 additional days (Data S1). Sirtinol treatment inhibited HDMEC long-term proliferation in a dose-dependent manner, with the concentrations of 5 and 10 µM reaching a significant level of inhibition at 9-days culture (Fig. S2A). In the subsequent experiments, a sirtinol dose of 10 µM was chosen. We next investigated the effect of sirtinol on HDMEC viability by analyzing apoptosis and necrosis. Measurement of cytoplasmic histone-associated DNA fragments revealed that sirtinol alone did not affect cell apoptosis or necrosis at 18 hours of treatment (data not shown). We also analyzed the effect of sirtinol on histone 3 (H3) and histone 4 (H4) total acetylation. In these experiments, to obtain a sufficient amount of histone proteins from the cell lysates to perform an enzyme-linked immunosorbent assay (ELISA), an immortalized human microvascular endothelial cell line was used (Data S1). As shown in Fig. S2B, sirtinol treatment resulted into increased acetylation of both H3 and H4. The effect of sirtinol on histone acetylation grade was then confirmed by immunofluorescence on primary HDMEC. As shown in Fig. S2C, deacetylation of H4 lysine 16 residue, a preferential substrate for Sirt1 [8], was highly inhibited, and deacetylation of H3 lysine 9 residue was also impaired, both leading to augmented histone acetylation.

### Sirtinol treatment regulates cellular responses to inflammatory stimuli

**Figure 2:**
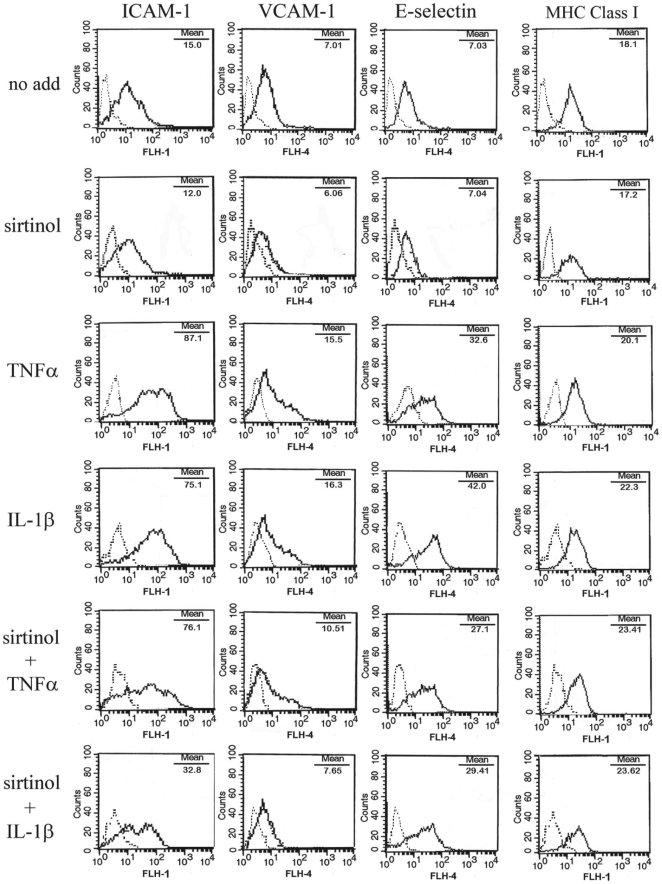
Expression of adhesion molecules in sirtinol-treated HDMEC after a 5-hour stimulation. HDMEC were analyzed for ICAM-1, VCAM-1, E-selectin, and MHC Class I by flow cytometry after 18-hour treatment with medium alone (no add) or 10 µM sirtinol, followed by a 5-hour stimulation with 10 ng/ml of the indicated cytokines. Dotted lines represent staining with matched isotype Ig. The x-axis and the y-axis indicate the relative cell number and mean fluorescence intensity, respectively. Data are representative of at least ten different experiments with similar results.

**Figure 3:**
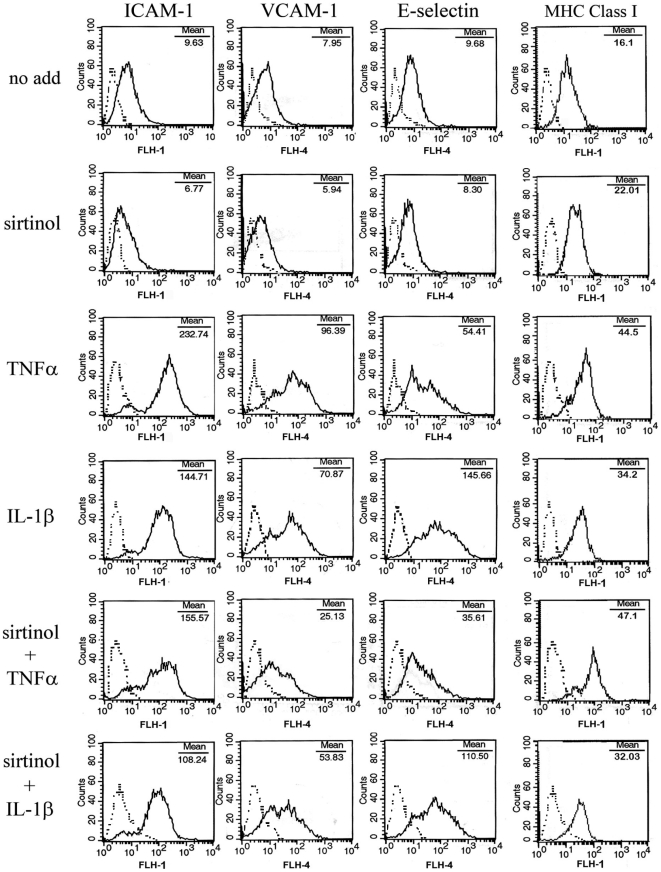
Expression of adhesion molecules in sirtinol-treated HDMEC after a 24-hour stimulation. HDMEC were analyzed for ICAM-1, VCAM-1, E-selectin, and MHC Class I expression by flow cytometry after treatment with medium alone (no add) or sirtinol, followed by a 24-hour stimulation with the indicated cytokines. Dotted lines represent staining with matched isotype Ig. The x-axis and the y-axis indicate the relative cell number and mean fluorescence intensity, respectively. Data are representative of at least twelve different experiments with similar results.

To analyze the effect of sirtinol on HDMEC response to inflammatory stimuli, HDMEC were sequentially treated with sirtinol and/or with 10 ng/ml tumor necrosis factor (TNF)α or interleukin (IL)-1β, two cytokines having a potent pro-inflammatory activity on endothelial cells [26]. After 5-hour or 24-hour treatments, HDMEC were examined by flow cytometry (FACS) for expression of inflammation-related adhesion molecules. We found that sirtinol alone did not modify membrane expression of the examined proteins but, after 5-hour treatment with TNFα, it slightly reduced expression of ICAM-1, VCAM-1, and E-selectin (Fig. 2). After 24-hour stimulation with TNFα, sirtinol pretreatment significantly reduced membrane expression of ICAM-1, VCAM-1, and E-selectin, without affecting MHC class I (Fig. 3). Upon stimulation with IL-1β for 5 hours in the presence of sirtinol, a clear-cut down-regulation of ICAM-1, VCAM-1, and E-selectin expression was observed (Fig. 2). After 24-hour treatment with IL-1β, sirtinol led to a significant reduction of ICAM-1, VCAM, and E-selectin membrane levels, although less marked than those obtained with TNFα treatment (Fig. 3). Again, MHC class I expression was not modified by sirtinol pretreatment (Fig. 3).

**Figure 4:**
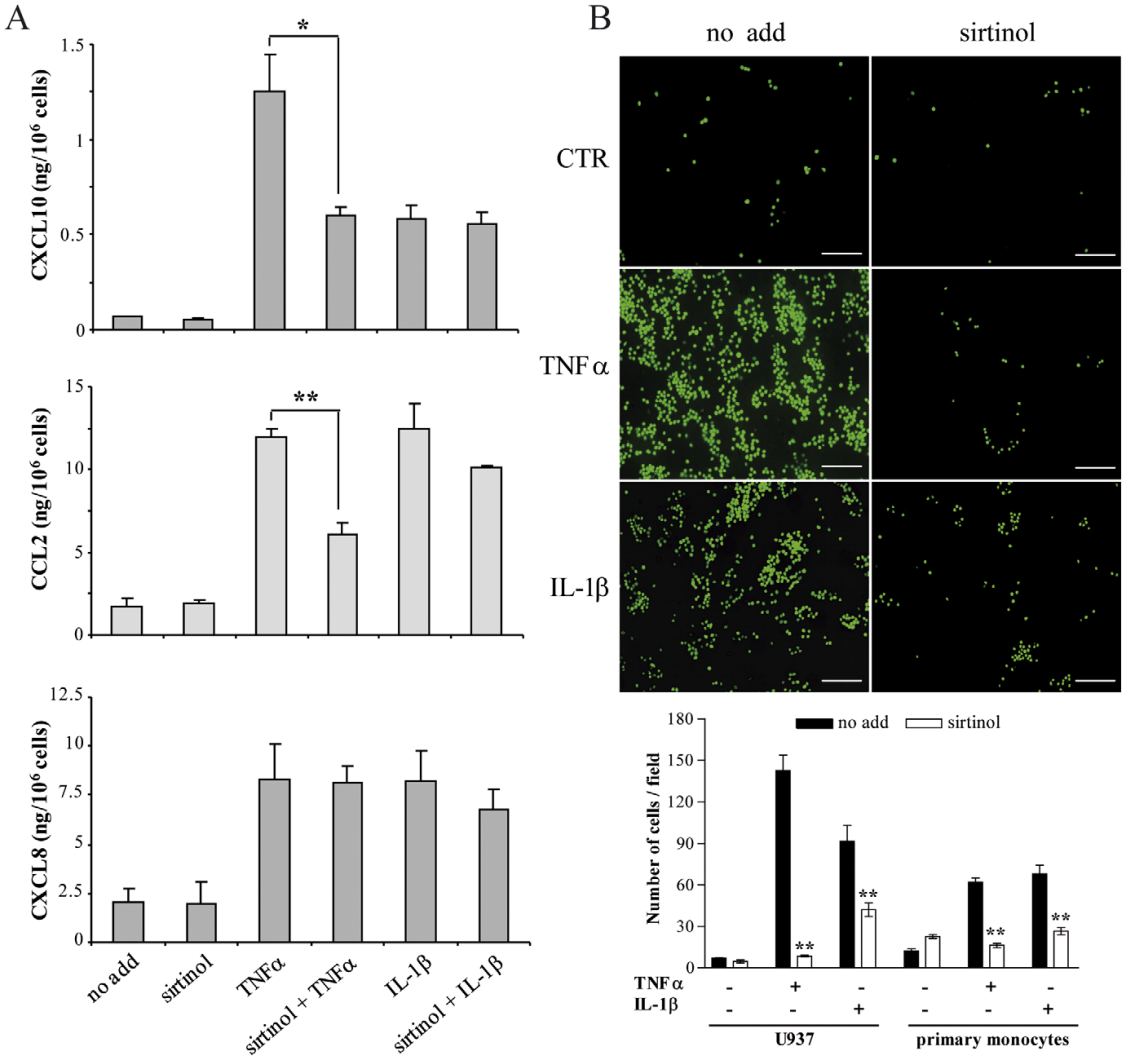
Chemokine secretion and monocyte adhesion in sirtinol-treated HDMEC. (A) HDMEC conditioned medium was analyzed by ELISA after treatment with medium alone (no add) or sirtinol, followed by stimulation with the indicated cytokines. Results are the mean of at least three independent experiments and are given in ng/10^6^ cells ± s.d; *p≤0.01, **p≤0.001, T-student test. (B) Fluorescence-labelled U937 cells or primary monocytes were plated on HDMEC pretreated or not (no add) with sirtinol, and exposed or not (CTR) to TNFα or IL-1β. Adherent cells were quantified as the mean number of fluorescent cells present in 10 randomly selected fields for each condition. Pictures of a representative experiment are shown in the left panels, bar  =  50 µm. Data are expressed as the mean of three different experiments ± s.d (panel below), **p≤0.005, T-student test.

Conditioned medium of HDMEC treated with sirtinol and with TNFα or IL-1β was then examined by ELISA for vascular endothelial growth factor-A (VEGF-A) and for inflammatory chemokine secretion. In basal conditions, HDMEC did not secrete detectable amounts of VEGF-A and sirtinol treatment, alone or in combination with TNFα or IL-1β, did not induce any further production of the growth factor (data not shown). CXCL10, CXCL8, and CCL2 secretion was not significantly modified in HDMEC treated with sirtinol, either in respect to basal condition or compared with cells concurrently treated with IL-1β for 24 hours (Fig. 4A). However, sirtinol pretreatment significantly inhibited the secretion of CXCL10 and CCL2, but not of CXCL8, induced by a 24-hour treatment with TNFα (Fig. 4A).

Down-regulation of adhesion molecule or chemokine expression observed in sirtinol- and TNFα- or IL-1β-treated HDMEC was not due to a modulation of the receptors for TNFα or IL-1β, in particular of IL-1 receptor I and TNF receptor 1, since their expression was not affected by sirtinol, as demonstrated by RT-PCR analysis of untreated and 18-hour sirtinol-treated samples (data not shown).

To investigate the biological significance of sirtinol-mediated reduction of expression of cell surface immunomodulatory molecules and of release of inflammatory chemokines potentially involved in the recruitment of leukocytes during inflammation, a monocyte adhesion assay on activated HDMEC was performed. The U937 cell line was chosen for the initial studies, as it exhibits many characteristics of primary monocytes and it has been widely used as a model for blood-borne monocytes [27]. Very few fluorescence-labelled U937 cells adhered to HDMEC over 2 hours in the absence of TNFα or IL-1β stimulus, regardless to the previous exposure to sirtinol (Fig. 4B). On the other hand, after HDMEC stimulation with the two inflammatory cytokines, the number of adherent cells greatly increased and was dramatically reduced when HDMEC were pretreated with sirtinol (Fig. 4B). Adhesion assays on activated HDMEC were then repetead using fresly isolated human primary monocytes, and gave comparable results (Fig. 4B).

### Sirt1 is involved in HDMEC inflammatory responses

**Figure 5:**
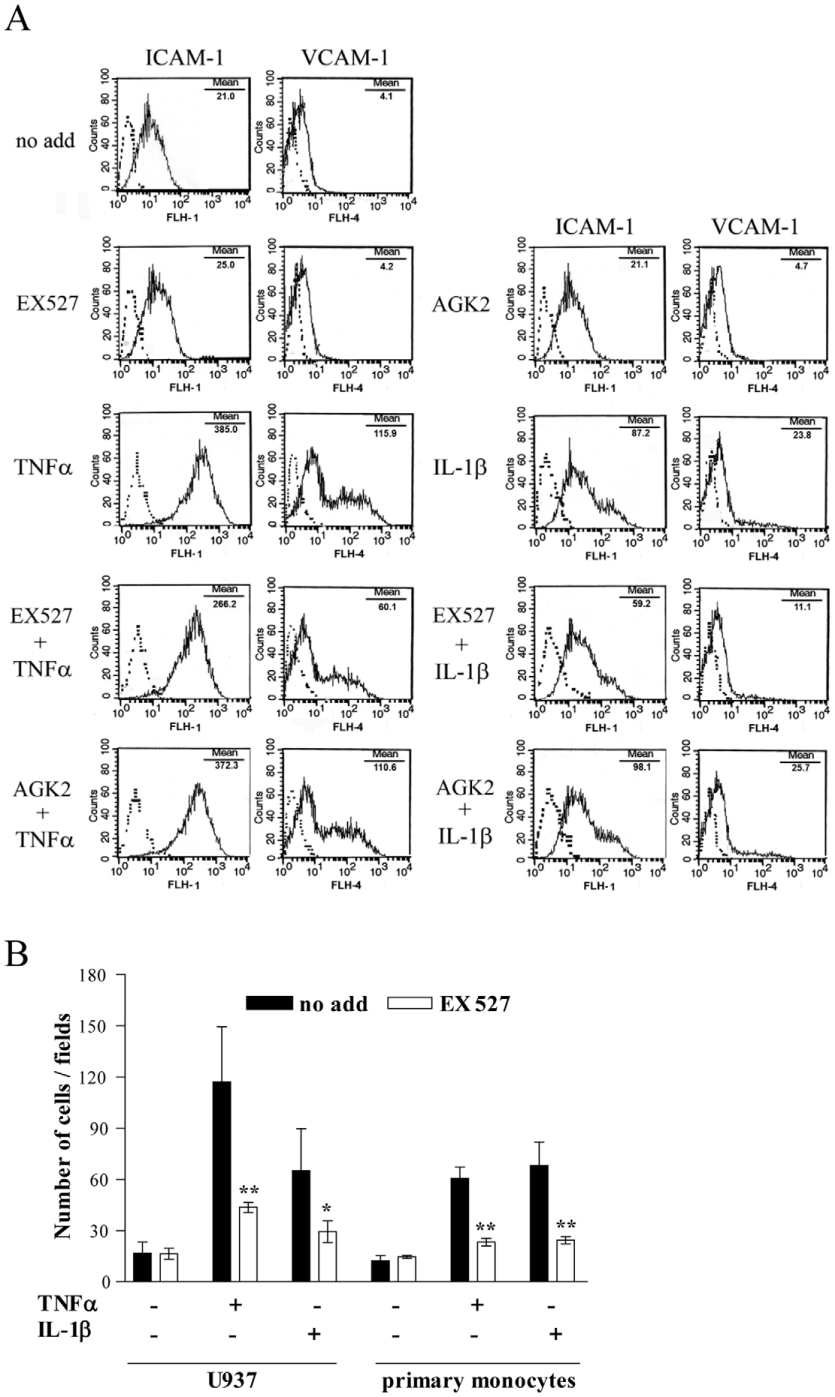
Adhesion molecule expression and monocyte adhesion in EX527- or AGK2-treated HDMEC. (A) HDMEC were analyzed for ICAM-1 and VCAM-1 expression by flow cytometry after treatment with medium alone (no add), EX527, or AGK2, followed by a 5-hour stimulation with the indicated cytokines. Dotted lines represent staining with matched isotype Ig. The x-axis and the y-axis indicate the relative cell number and mean fluorescence intensity, respectively. Data are representative of three different experiments which gave similar results. (B) Fluorescence-labelled U937 cells or primary monocytes were plated on HDMEC pretreated or not (no add) with EX527, and exposed or not (CTR) to TNFα or IL-1β. Adherent cells were quantified as the mean number of fluorescent cells present in 10 randomly selected fields for each condition. Data are expressed as the mean of three different experiments ± s.d; *p≤0.05, **p≤0.005, T-student test.

Sirtinol inhibitory properties have been tested *in vitro* on human Sirt1 and Sirt2 [25]. Therefore, in an attempt to identify which sirtuin was mainly involved in the modulation of HDMEC inflammation responses, we used the two inhibitors, 6-chloro-2,3,4,9-tetrahydro-1H-carbazole-1-carboxamide or EX527 [28], and 2-cyano-3-[5-(2,5-dichlorophenyl)-2-furanyl]-N-5-quinolinyl-2-propenamide or AGK2 [29], selectively affecting Sirt1 and Sirt2, respectively. FACS analysis showed that Sirt1 blocking reduced ICAM-1 and VCAM expression induced by both TNFα and IL-1β after the 5-hour-treatment (Fig. 5A) and, to a lesser extent, after the 24-hour one (data not shown). On the other hand, inhibition of Sirt2 did not decrease the expression of ICAM-1 and VCAM after treatment with IL1β and TNFα (Fig. 5A). As no anti-inflammatory effect was detected following AGK2 treatment, we verified that the selected dose of AGK2 was effectively inhibiting Sirt2 in HDMEC. We analyzed tubulin acetylation as it represents the main target of Sirt2 deacetylase activity (Data S1) and found a 2.5 time increase in tubulin acetylation following AGK2 treatment (Fig. S1C).

Finally, to prove that the observed EX527-mediated reduction of cell surface adhesion molecules was sufficient to sustain an anti-inflammatory effect in HDMEC, the monocyte adhesion assay was repeated. As shown in Fig. 5B, after HDMEC stimulation with the two inflammatory cytokines, the number of adherent monocytes, either U937 cell line or primary human monocytes, was significantly reduced when HDMEC were pretreated with EX527.

### Sirt1 is expressed in HDMEC in vivo

**Figure 6:**
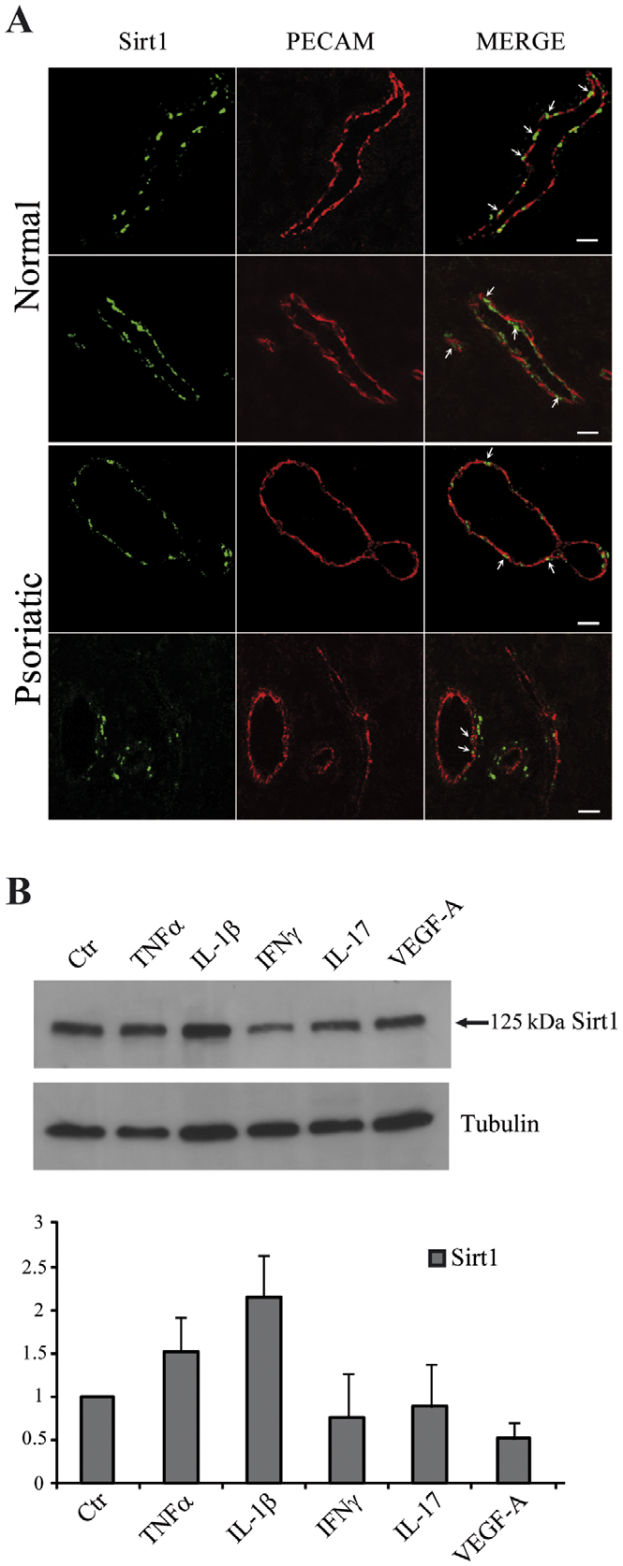
Sirt1 expression in skin microvessels. (A) Sirt1 was stained with the primary antibody (left panels), and endothelial cells were put in evidence with a co-staining for the PECAM/CD31 transmembrane adhesion molecule (middle panels). Merged pictures are in the right panels. Each panel is a total projection of a confocal stack of images; bars  =  20 µm. (B) HDMEC were treated with the indicated cytokines and the cell lysates were analyzed by immunoblotting with an anti-Sirt1 antibody. An antibody against tubulin was used as a loading control. A representative experiment is reported and the densitometric analysis of at least three different experiments is shown as the mean ± s.d.

To evaluate whether Sirt1 could represent a possible therapeutic target for the treatment of inflammatory skin diseases characterized by a prominent microvessel involvement, we analyzed Sirt1 expression *in vivo* in the microvessels of normal and psoriatic skin. As shown in Fig. 6A, dermal vessels clearly expressed Sirt1 in the endothelial cell nucleus. An analogous, even though less prominent staining, was also observed in the dermal vessels of psoriatic skin. To further investigate this down-modulation of Sirt1 expression in psoriatic HDMEC, we treated HDMEC *in vitro* for 24 hours with different cytokines, i.e. TNFα, IL-1β, interferon (IFN)-γ, IL-17, or VEGF-A, all highly present in the psoriatic skin lesions. Then, we analyzed by immunoblotting Sirt1 protein expression and observed that IFNγ, IL-17, and VEGF-A slightly, but not significantly, reduced Sirt1 amount in HDMEC (Fig. 6B).

## Discussion

Systematic investigation of sirtuin expression is an important preliminary step to study the selective role of class III HDAC. So far, this kind of studies has been mainly performed on transformed cells, and little is known about sirtuin expression in normal human cell types. Moreover, cell localization studies have been carried out through overexpression of exogenous sirtuins [30]. One sirtuin at the time was often examined, even though it has been reported that overexpression of a single exogenous sirtuin can interfere with localization of another one in a specific cellular compartment [21]. In the present work, we firstly evaluated the expression of endogenous sirtuins in HDMEC, a normal primary human cell type. Considering the mRNA production, Sirt1, Sirt2, Sirt5, and Sirt7 are expressed at higher levels as compared to Sirt3, Sirt4, and Sirt6. A similar but not entirely overlapping pattern could be observed in HUVEC, suggesting differences between micro and macrovascular endothelial cells, and between different skin cell types, such as keratinocytes, fibroblasts, and melanocytes. Protein expression and localization of four sirtuins were then evaluated, showing the expected nuclear localization for Sirt1 and the reported presence of Sirt7 in the nucleolar compartment. Interestingly, confocal microscopy and western blotting analysis of different cellular compartments detected Sirt2 both in the cytoplasm and in the nucleus, and Sirt5 both in mithocondria and in the nucleus. Sirt2 was previously reported to shuttle from the cytoplasmic to the nuclear compartment and vice versa, whereas Sirt5 localization in the nucleus was not previously shown. This data might reflect the existence of an additional nuclear target for the deacetylase activity of Sirt5 in HDMEC.

Then, to investigate the relevance of targeting sirtuins for the treatment of skin inflammatory diseases, we used the selective inhibitor sirtinol. As previously reported for HUVEC, we found that sirtinol reduced HDMEC long-term proliferation. Sirtinol treatment alone did not affect cell apoptosis and necrosis, showing absence of a significant basal toxicity at the used doses. Nevertheless, sirtinol treatment greatly inhibited histone deacetylation in HDMEC.

Importantly, we found that sirtinol short-term effects on HDMEC were mainly directed towards the down-modulation of the inflammatory response. Inhibitors of class I and II HDAC trigger both pro- and anti-inflammatory effects depending on the targeted cell type [31], [32]. Specifically regarding endothelial cells, treatment of HUVEC with trichostatin-A inhibited TNFα-mediated induction of VCAM-1, and effectively blocked VCAM-1-dependent leukocyte adhesion [33]. Likewise, we showed here that sirtinol pre-treatment reduces adhesion molecule expression and chemokine secretion induced by two inflammatory agents and block monocyte adhesion to activated endothelial cells. Our results clearly indicate that the overall final outcome of sirtinol treatment on HDMEC is the neutralization of exogenously-added inflammatory stimuli.

Using more selective inhibitors, we also showed that Sirt1 inhibition is largely involved in the anti-inflammatory action of sirtinol. In apparent contrast with our findings, Sirt1 was previously reported to suppress inflammation in several tissues [34], [35], [36]. In tumor cells, Sirt1 acts as a negative regulator of NF-κB, that represents a master regulator of inflammatory signalling [37]. However, Sirt1 knockout mice do not display the same inflammatory phenotype of mice expressing a constitutive active form of p65 NF-κB subunit [38], indicating that Sirt1 does not modulate the totality of NF-κB-dependent genes. Moreover, trichostatin-A, that exerts an anti-inflammatory effect on endothelial cells, was reported to potentiate NF-κB-dependent transcription in tumor cells [37]. Clearly, more complex regulatory processes are present in the onset of inflammation and are expected to be cell-type specific [32].

In addition, our present data and the indication of another study on the anti-inflammatory effect of sirtinol [14], strongly suggest that a broad inhibition of sirtuins could act differently from the selective interference of one single enzyme. Indeed, when we used the Sirt1 selective inhibitor EX527, we did not reach the same anti-inflammatory effect obtained with sirtinol, clearly indicating the existence of a multifaceted sirtuin network acting in response to the inflammatory stimuli.

Finally, we showed that Sirt1 was expressed *in vivo* in normal and psoriatic human skin. Interestingly, Sirt1 expression was reduced in the nuclei of the psoriatic dermal vessels. This could be due to the effect of cytokines, such as the lymphokine IFN-γ, abundantly present in the psoriatic lesion. Indeed, Sirt1 levels were reduced also in the basal keratinocytes of psoriatic lesions, with IFN-γ inhibiting Sirt1 expression [39]. However, treatment of HDMEC *in vitro* with IFN-γ, as well as with IL-17 or VEGF-A, slightly and not significantly reduced Sirt1 protein expression, indicating that the observed down-modulation of Sirt1 expression observed *in vivo* could not be due to a direct action of a single inflammatory cytokine. In addition, the endogenous down-modulation of Sirt1 expression in psoriatic skin *per se* is not sufficient to suppress local inflammatory responses and a further reduction of Sirt1 could be beneficial in a therapeutic treatment.

In conclusion, our study highlights previously unrecognized anti-inflammatory properties of sirtinol in dermal endothelial cells, and suggests that sirtuins could represent potential targets for the treatment of skin inflammation.

## Materials and Methods

### Cell culture and reagents

HDMEC were isolated from foreskin of 4 different donors as previously described [40], cultured in EGM-2MV (Lonza, Basel, Switzerland), and used at passage 4 to 8. HUVEC were isolated from 3 different donors, cultured as previously described [41], and used up to passage 4. To obtain primary human monocytes, peripheral blood mononuclear cells (PBMC) were separated from healthy donor whole blood by density gradient centrifugation over Lymphoprep (Nycomed-Pharmacia, Oslo, Norway). After extensive washing, PBMC were separated by immunomagnetic positive selection into CD14^+^ population by using magnetic beads according to the manufacturer's protocol (Miltenyi Biotec, Gladbach, Germany) and resulting in a 90% to 98% monocyte purity. U937 cell line (American Type Culture Collection, ATCC, LGC Standards, UK) was maintained in RPMI-1640 medium (Life Technologies, Grand Island, NY), supplemented with 10% fetal calf serum (Lonza) and 2 mM/l L-glutamine (Life Technologies). HeLa cell line (ATCC) was grown and maintained in DMEM medium (Life Technologies) supplemented with 10% fetal calf serum and 2 mM/l L-glutamine.

Recombinant human TNFα, IL-1β, VEGF-A, IFNγ and IL-17 were purchased from R&D Systems (Minneapolis, MN). Sirtinol was prepared according to reported procedures [25]. EX527 and AGK2 were purchased from Tocris Bioscience, Bristol, UK.

### RT-PCR

Cells were processed for RNA extraction utilizing the RNeasy Plus Mini Kit (Qiagen, Hilden, Germany) according to supplier's instructions. Reverse transcription was performed with Superscript III RT (Invitrogen, Carlsbad, CA) starting from 1 µg of total RNA. Primer sequences were as follows: Sirt1 (GenBank NM_012238; oligonucleotide forward 405-428 bp, reverse 563-586 bp); Sirt2 (GenBank NM_012237; oligonucleotide forward 438–461 bp, reverse 699–722 bp); Sirt3 (GenBank NM_012239; oligonucleotide forward 290–313 bp, reverse 527–550 bp); Sirt4 (GenBank NM_012240; oligonucleotide forward 414–437 bp, reverse 565–588 bp); Sirt5 (GenBank NM_012241; oligonucleotide forward 365–388 bp, reverse 507–530 bp); Sirt6 (GenBank NM_016539; oligonucleotide forward 301–324 bp, reverse 487–510 bp); Sirt7 (GenBank NM_016538; oligonucleotide forward 605–628 bp, reverse 849–872 bp). Amplification conditions were: denaturation 95°C, 30 sec; annealing 55°C, 1 min; extension 68°C,1 min. [α-32P]dATP was added to the reaction (0.04 µCi/µl); 32 cycles were performed to obtain sirtuin amplicons, whereas 24 cycles were used for GAPDH amplicon. PCR products were separated on a 6% polyacrylamide gel and quantitatively analyzed in a Typhoon Trio imager system using the IMAGE QUANT version 5.0 software (GE Healthcare, Amersham, UK). PCR products were also separated on a 2% agarose gel, extracted from the gel with the Gel Extraction kit (Qiagen) and sequenced.

### Western blotting

Confluent HDMEC were lysed in a buffer containing 20 mM Tris-HCl, 150 mM NaCl, 1% Triton. Cell lysates were centrifuged, and supernatants directly evaluated for total protein amount with the BioRad Protein Assay (BioRad, Hercules, CA). Equal quantity of total proteins were separated by 6 or 12% SDS-gel electrophoresis and transferred onto a nitrocellulose membrane (Hybond-ECL, GE Healthcare). Protein detection was performed using the following antibodies: Sirt1 (H-300) and Sirt2 (A-5) (Santa Cruz Biotechnologies, Santa Cruz, CA), Sirt5 (Enzo Life Science, Plymouth Meeting, PA), Sirt7 (Sigma, Saint Louis, MO), α-tubulin (Calbiochem, Darmstadt, Germany), GAPDH (Santa Cruz Biotechnologies), and a chemiluminescence detection system (GE Healthcare). Relative intensity of signals was quantified using a GS-710 densitometer (Bio-Rad). In selected experiments, HDMEC were treated with 10 ng/ml TNFα, 10 ng/ml IL-1β, 30 ng/ml VEGF-A, 500 U/ml IFNγ, or 20 ng/ml IL-17 for 24 hours. Equal quantity of total protein lysates were separated by 4–15% SDS-gradient gel electrophoresis and transferred onto a nitrocellulose membrane. Protein detection and loading normalization were performed using the anti-Sirt1 antibody H-300 (Santa Cruz Biotechnologies) and the anti-α-tubulin antibody (Calbiochem), respectively.

### Immunofluorescence and confocal analysis

HDMEC were seeded on coverslips, synchronized by serum deprivation, and allowed to reach subconfluence. For mitochondria staining, cells were washed with 1x phosphate buffer (PBS) and incubated with 40 nM MitoTracker® Red (CMXRos, Life Technologies) for 45 min at 37°C. Cells were then fixed with 3% paraformaldehyde/PBS, permeabilized with 0.1% Triton X-100, and blocked for 30 min with 1% BSA, followed by incubation with primary antibody overnight at 4°C. Antibodies were as follows: Sirt1 (D739, Cell Signaling Technology, Beverly, MA), Sirt2 (Epitomics, Burlingame, CA), Sirt5 (Enzo Life Science and Abnova, Taipei City, Taiwan), Sirt7 [22], UBF (F-9, Santa Cruz Biotechnologies). After washing in 1x PBS, cells were incubated with the appropriate secondary antibodies 1 hour at room temperature. Nuclei were visualized by adding 1 µM TO-PRO®-3 iodide (Life Technologies) for 10 min at room temperature. For tissue studies, four-µm-thick paraformaldehyde-fixed, paraffin-embedded human skin sections were used, and specimens were deparaffinized, rehydrated, and processed as previously described [42]. Tissue was permeabilized with 0.1% Triton X-100. An anti-Sirt1 polyclonal antibody (E104, Abcam) was used, at a dilution of 1∶100 and incubated over night at +4°C. The signal was amplified with the TSA biotin system (Perkin-Elmer Life Science, Boston, MA) following the manufacturer's instructions. To identify endothelial cells, an anti-human PECAM/CD31 polyclonal antibody (JC/70A, Abcam) was used, diluted 1∶25 and incubated for 1 hour at 37°C. After washing, sections were incubated with the appropriate secondary antibodies 1 hour at room temperature. Negative controls were performed by omitting the primary antibody. Coverslips were mounted and slides analyzed using a Zeiss LSM 510 meta-confocal microscope (Zeiss, Oberkochen, Germany).

### FACS analysis

HDMEC were cultured to subconfluence, starved of serum and growth factors for 6 hours, treated with 10 µM sirtinol for 18 hours, and stimulated with 10 ng/ml IL-1β or TNFα for 5 or 24 hours. In selected experiments, serum-starved HDMEC were treated with 1 µM EX527 or 10 µM AGK2 for 18 hours before stimulation with IL-1β or TNFα. Expression of membrane ICAM-1, VCAM, E-selectin, and MHC class I was evaluated after 5 and 24 hours using fluorescein isothiocyanate (FITC)-conjugated anti-ICAM-1 (clone 84H10, Immunotech, Marseille, France), anti-MHC class I (clone G46 2.6, BD Pharmingen, Franklin Lakes, NJ), allophycocyanin (APC)-conjugated anti-human VCAM-1 (clone 51-10C9, BD Pharmingen) and anti-human E-selectin (clone 68-5H11, BD Pharmingen). In control samples, staining was performed using isotype-matched control antibodies. Morphological features and parameters of cells treated with sirtinol and/or cytokines were similar. Cells were analyzed with a FACScan equipped with Cell Quest software (Becton Dickinson, Mountain View, CA).

### ELISA

Cell-free supernatants from unstimulated or stimulated HDMEC were tested for VEGF-A amount, using the Quantikine kit (R&D Systems), and for CXCL10, CCL2, and CXCL8 content, using OptEIA kits (BD Pharmingen), following manufacturer's protocol. Plates were analyzed in a Microplate reader 3550-UV (BioRad). HDMEC supernatants were assayed in triplicate for each condition.

### Monocyte adhesion to HDMEC

U937 cell line or primary human monocytes were fluorescently labeled with 2′,7′-bis-(2-carboxyethyl)-5-(and-6)-carboxy-fluorescein acetoxymethyl ester (BCECF-AM, Molecular Probes, Eugene, OR) as described [43]. Briefly, cells (1×10^7^ cells/5 ml) were incubated with 5 µM/l BCECF-AM in RPMI-1640 medium for 30 min at 37°C. Cells were then washed three times with 1x PBS to remove excess dye and resuspended at a density of 5×10^5^ cells/ml. HDMEC were cultured to confluence on coverslips and treated at 37°C with 10 µM sirtinol or with 1 µM EX527 for 18 hours. HDMEC were then stimulated with 10 ng/ml IL-1β or TNFα for additional 24 hours. BCECF-labeled U937 and BCECF-labeled primary monocytes (2.5×10^5^ cells/well) were incubated with treated and untreated HDMEC for 120 min at 37°C. Non-adherent cells were removed by washing with 1x PBS. Cells were fixed with 3% paraformaldehyde/PBS, washed in 1x PBS, and mounted on slides. At least ten randomly selected fields for each treatment were recorded with a digital camera (AxioCam MRc5, Zeiss) and fluorescent cells were counted.

## Supporting Information

Figure S1 **Sirtuin RNA and protein expression in skin cell types and effect of sirtuin inhibition on tubulin acetylation.** (A) Semi-quantitative RT-PCR analysis of sirtuin expression in normal primary human fibroblasts (FB), keratinocytes (K), and melanocytes (Mel). Bars refer to sirtuin mRNA amounts relative to GAPDH mRNA. Results are shown as the mean ± s.d. of at least three experiments performed on cells isolated from different individuals. (B) Sirt2 and Sirt5 subcellular localization was analyzed by Western blotting in HDMEC whole extract (WE), nuclear (N), and cytoplasmic (C) fraction that comprises also mitochondria. Sirt2 and Sirt5 polypeptides are indicated by arrows. The molecular weight of the sirtuin isoforms is given in kDa. Lamin A/C is a nuclear protein, tubulin is present in the cytoplasm, and Cox4 is a mitochondria specific protein, all used to verify protein separation in the different cell compartments. A representative experiment is shown. (C). Western blotting analysis of acetyl-tubulin (upper panel) or total tubulin (lower panel) in endothelial cells not treated or treated for 18 hours with the selective Sirt1 inhibitor (EX527) or the specific Sirt2 inhibitor (AGK2). Molecular weight of acetyl-tubulin is given in kDa. A representative experiment is reported. The densitometric analysis of two different experiments is shown as the mean ± s.d.
(TIF)

Figure S2 **Effect of sirtinol on HDMEC proliferation and histone acetylation.** (A) HDMEC were treated with different concentrations of sirtinol or left untreated (no add). Cell proliferation was measured, at the indicated days after stimulus, by cell staining with crystal violet and determination of the A_540_. Results are shown as the mean ± s.d. of at least three independent experiments; *p≤0.005, ** *p*≤0.01. (B) HMEC cells were treated with 10 µM sirtinol (sirtinol) or left untreated (no add). Total acetylation level of H3 and H4 was assessed by determining A_450_ as described in Data S1. Absorbance in non specific wells is also reported (blank). Results are expressed as the mean of two different experiments ± s.d. (C) HDMEC were treated with 10 µM sirtinol (Sirtinol) or left untreated (No add). Cells were stained with antibodies against H4 acetylated lysine 16 (H4K16acetyl, left panel) and H3 acetylated lysine 9 (H3K9acetyl, right panel). Dapi was used to counter stain nuclei in all experiment. Bar =  10 µm.
(TIF)

Data S1 **Methods for human skin primary cell and HMEC cell line culture.** Methods for preparation of nuclear and cytoplasmic fractions from a HDMEC whole cell lysate and subsequent western blotting assay. Methods for analysis of HDMEC proliferation, and of HMEC and HDMEC histone or tubulin acetylation status.
(DOC)
