# Correction: Sirtinol Treatment Reduces Inflammation in Human Dermal Microvascular Endothelial Cells

**DOI:** 10.1371/annotation/0174b439-c62b-42e8-a420-3defbfe5c8e9

**Published:** 2011-10-31

**Authors:** Angela Orecchia, Claudia Scarponi, Francesca Di Felice, Elisa Cesarini, Simona Avitabile, Antonello Mai, Maria Luisa Mauro, Valentina Sirri, Giovanna Zambruno, Cristina Albanesi, Giorgio Camilloni, Cristina M. Failla

The authors wish to add the following funding source to the financial disclosure information: "E.C. is recipient of the "Teresa Ariaudo Fellowship" from Istituto Pasteur-Fondazione Cenci Bolognetti". 

