# Supplementary figures and images for: Sirtinol Treatment Reduces Inflammation in Human Dermal Microvascular Endothelial Cells
Source: PLoS One. 2011 Sep 12;6(9):e24307. doi: 10.1371/journal.pone.0024307 (PMC3171404; doi:10.1371/journal.pone.0024307)

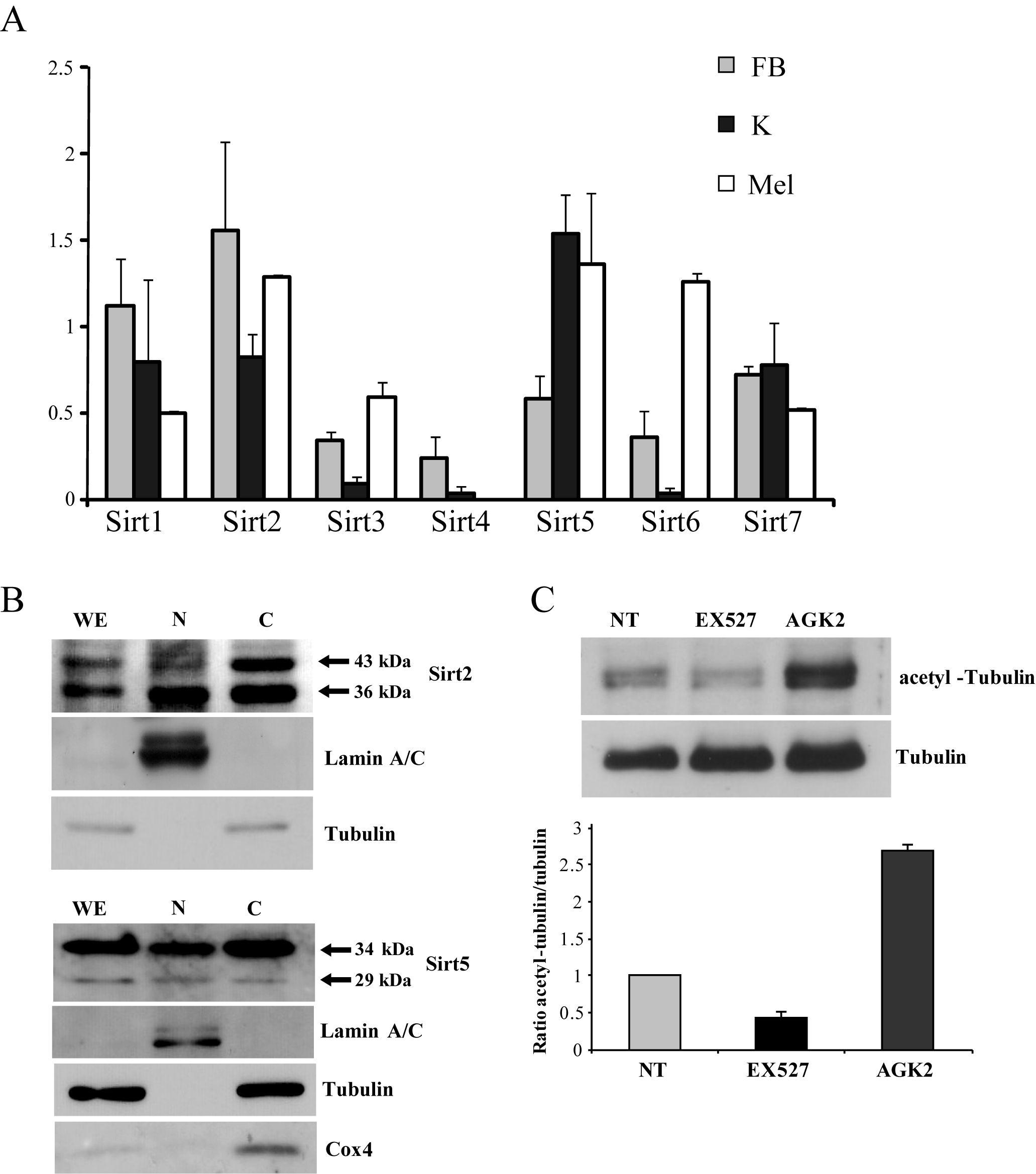

Supplement: Figure S1 — Sirtuin RNA and protein expression in skin cell types and effect of sirtuin inhibition on tubulin acetylation. (A) Semi-quantitative RT-PCR analysis of sirtuin expression in normal primary human fibroblasts (FB), keratinocytes (K), and melanocytes (Mel). Bars refer to sirtuin mRNA amounts relative to GAPDH mRNA. Results are shown as the mean ± s.d. of at least three experiments performed on cells isolated from different individuals. (B) Sirt2 and Sirt5 subcellular localization was analyzed by Western blotting in HDMEC whole extract (WE), nuclear (N), and cytoplasmic (C) fraction that comprises also mitochondria. Sirt2 and Sirt5 polypeptides are indicated by arrows. The molecular weight of the sirtuin isoforms is given in kDa. Lamin A/C is a nuclear protein, tubulin is present in the cytoplasm, and Cox4 is a mitochondria specific protein, all used to verify protein separation in the different cell compartments. A representative experiment is shown. (C). Western blotting analysis of acetyl-tubulin (upper panel) or total tubulin (lower panel) in endothelial cells not treated or treated for 18 hours with the selective Sirt1 inhibitor (EX527) or the specific Sirt2 inhibitor (AGK2). Molecular weight of acetyl-tubulin is given in kDa. A representative experiment is reported. The densitometric analysis of two different experiments is shown as the mean ± s.d. (TIF) [file pone.0024307.s001.tif]

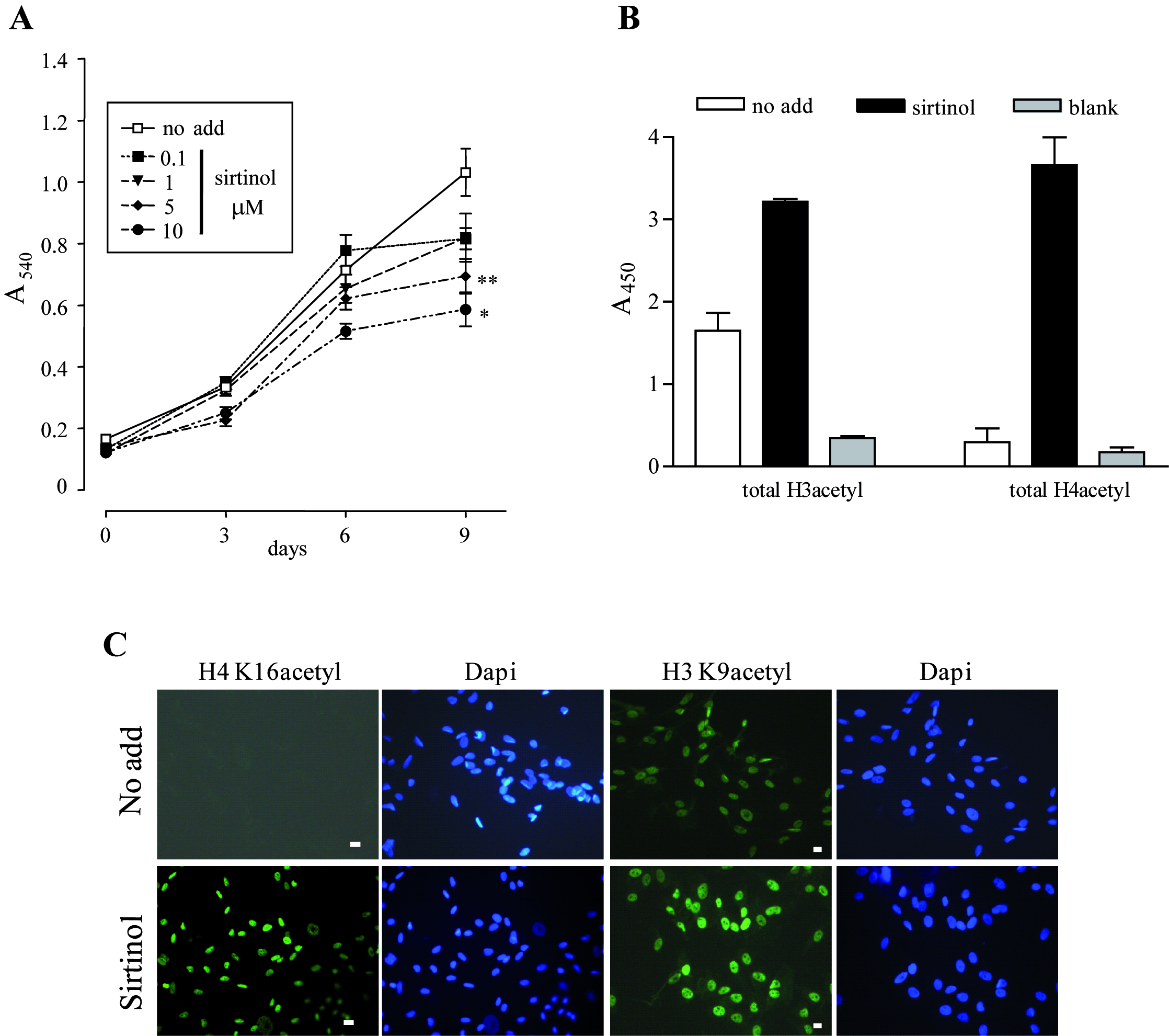

Supplement: Figure S2 — Effect of sirtinol on HDMEC proliferation and histone acetylation. (A) HDMEC were treated with different concentrations of sirtinol or left untreated (no add). Cell proliferation was measured, at the indicated days after stimulus, by cell staining with crystal violet and determination of the A540. Results are shown as the mean ± s.d. of at least three independent experiments; *p≤0.005, ** p≤0.01. (B) HMEC cells were treated with 10 µM sirtinol (sirtinol) or left untreated (no add). Total acetylation level of H3 and H4 was assessed by determining A450 as described in Data S1. Absorbance in non specific wells is also reported (blank). Results are expressed as the mean of two different experiments ± s.d. (C) HDMEC were treated with 10 µM sirtinol (Sirtinol) or left untreated (No add). Cells were stained with antibodies against H4 acetylated lysine 16 (H4K16acetyl, left panel) and H3 acetylated lysine 9 (H3K9acetyl, right panel). Dapi was used to counter stain nuclei in all experiment. Bar = 10 µm. (TIF) [file pone.0024307.s002.tif]
