# Supplementary material for: Sirtinol Treatment Reduces Inflammation in Human Dermal Microvascular Endothelial Cells
Source: PLoS One. 2011 Sep 12;6(9):e24307. doi: 10.1371/journal.pone.0024307 (PMC3171404; doi:10.1371/journal.pone.0024307)
Supplement: Data S1 — Methods for human skin primary cell and HMEC cell line culture. Methods for preparation of nuclear and cytoplasmic fractions from a HDMEC whole cell lysate and subsequent western blotting assay. Methods for analysis of HDMEC proliferation, and of HMEC and HDMEC histone or tubulin acetylation status. (DOC) [file pone.0024307.s003.doc]

**Supplemental data**

**Methods**

***Cell culture****.*Normal human keratinocytes were obtained from foreskin of healthy males [1], and cultured in keratinocyte growth medium (KGM, Lonza, Basel Switzerland). Normal human fibroblasts were isolated from human skin biopsies and cultured as previously described [2]. Normal human melanocytes were isolated from human skin biopssies as previously described [3].

HMEC cell line was a generous gift of Prof. Candal, Centers for Diseases Control, Atlanta, GA, and was cultured as described [4].

***Subcellular localization of sirtuins.*** To prepare whole cell lysates, confluent HDMEC were suspended in a lysis buffer containing 20 mM Tris-HCl, 150 mM NaCl, 1% Triton. To separate the nuclear and the cytoplasmic fractions, the Nuclear Extract kit (Active Motif, Carlsbad, CA) was used as indicated by the manufacturer. Each protein sample was assayed for total protein quantification with the BioRad Protein Assay (BioRad, Hercules, CA). Equal amount of total proteins were separated by 12% SDS-gel electrophoresis and transferred to a nitrocellulose filter (Hybond-ECL, GE Healthcare, Fairfield, CT). Protein analysis was sequentially performed on the same filter using the following antibodies: Sirt2 (A-5, Santa Cruz Biotechnologies), α-tubulin (Calbiochem, Darmstadt, Germany), lamin A/C (Santa Cruz Biotechnologies); or Sirt5 (Enzo Life Science, Plymouth Meeting, PA, and Abnova, Taipei City, Taiwan), α-tubulin, lamin A/C, and Cox 4 (20E8, Santa Cruz Biotechnology). Detection was done with a chemiluminescence detection system (GE Healthcare, Amersham, UK).

***Proliferation assay.*** HDMEC were seeded at a concentration of 1x10^4^cells/well in a 96 well-culture plate, starved in the absence of serum and growth factors for 6 hours, and treated with different sirtinol concentrations for 18 hours at 37°C. At the end of the treatment, fresh medium supplemented with 10% FCS was added. At day 3, 6, and 9, cells were washed with PBS and then fixed with 3% formaldehyde and stained with 0.5% crystal violet. Dye absorbance at 540 nm was measured in a Microplate reader 3550-UV (BioRad).

***Histone acetylation analysis.*** HMEC cell line (50x10^6^ cells for each condition) were treated with sirtinol or left untreated for 16 hours. Cells were lysed with PBS containing 0.5% Triton X 100 and nuclei were separated by centrifugation. Acid-soluble proteins were isolated from nuclei in 200 μl of 0.5 N HCl at 4°C for 1 hour. After centrifugation, histones were precipitated from the supernatant in 1 ml of acetone and placed at -20°C overnight. After centrifugation, the proteins were re-suspended in 50 μl of H_2_O. Histone proteins (2 μg) were analyzed using the EpiQuik™ Global Acetyl Histone Quantification Kit (Epigentek Group Inc., Brooklyn, NY) for H4 and H3, according to the manufacture’s instruction. Absorbance was determined at 450 nm using a Microplate reader 3550-UV (Bio-Rad, Hercules, CA)

In different experiments, HDMEC were seeded on coverslips, allowed to reach confluence, and treated with sirtinol or left untreated for 16 hours. Cells were then fixed with 3% paraformaldehyde/PBS, permeabilized with 0.1% Triton X-100, and blocked for 30 min with 1% BSA, followed by incubation with antibodies against H4 acetylated lysine residue 16 or H3 acetylated lysine residue 9 (Millipore, Billerica, MA).

***Tubulin acetylation analysis.*** HDMEC were treated with 1 mM EX-527 or 10 mM AGK2 for 6 hours or left untreated. Cells were lysed in a 50 mM Tris, pH 7.8, 137 mM NaCl, 10 mM NaF, 1 mM EDTA, 1% Triton X-100, 1 mM dithiothreitol, 10% glycerol buffer containing Complete Mini protease inhibitor cocktail tablets (Roche, Basel, Switzerland) and supplemented with 10 μM TSA and 5 mM nicotinamide to prevent deacetylation after cell lyses [5,6]. Cell lysates were centrifuged, and supernatants directly evaluated for total protein amount with the BioRad Protein Assay (BioRad). Ten micrograms of total proteins were separated by 10% SDS-gel electrophoresis and transferred onto a nitrocellulose filter (Hybond-ECL, GE Healthcare). Protein detection was performed using the anti-acetyl-tubulin mouse monoclonal antibody (1:1000, SIGMA), the appropriate secondary antibody and a chemiluminescence detection system (GE Healthcare). The same filter was then probed with the anti-tubulin mouse monoclonal antibody (1:1000, Calbiochem). Relative intensity of signals was quantified using a GS-710 densitometer (Bio-Rad).

**References**

1. Zambruno G, Marchisio P, Marconi A, Vaschieri C, Melchiori A, et al. (1995) Transforming growth factor-β1 modulates β1 and β5 integrin receptors and induces de novo expression of the αvβ6 heterodimer in normal human keratinocytes: implications for wound healing. J Cell Biol 129: 853-865.

2. Wirtz MK, Glanville RW, Steinmann B, Rao VH, Hollister DW (1987) Ehlers-Danlos syndrome type VIIB. Deletion of 18 amino acids comprising the N-telopeptide region of a pro-alpha 2 (I) chain. J Biol Chem 262: 16376-16385.

3. De Luca M, D'Anna F, Bondanza S, Franzi AT, Cancedda R (1988) Human epithelial cells induce human melanocyte growth in vitro but only skin keratinocytes regulate its proper differentiation in the absence of dermis. J Cell Biol 107: 1919-1926.

4. Ades EW, Candal FJ, Swerlick RA, George VG, Summers S, et al. (1992) HMEC-1: establishment of an immortalized human microvascular endothelial cell line. J Invest Dermatol 99: 683-690.

5. Outeiro TF, Kontopoulos E, Altmann SM, Kufareva I, Strathearn KE, et al. (2007) Sirtuin 2 inhibitors rescue a-synuclein-mediated toxicity in models od Parkinson's disease. Science 317: 516-519.

6. Solomon JM, Pasupuleti R, Xu L, McDonagh T, Curtis R, et al. (2006) Inhibition of SIRT1 catalytic activity increases p53 acetylation but does not alter cell survival following DNA damage. Mol Cell Biol 26: 28-38.
